# Paleo-biodegradation and hydrocarbon mixing in a major hybrid petroleum reservoir

**DOI:** 10.1038/s41598-023-31350-z

**Published:** 2023-03-16

**Authors:** Jaime Cesar, Omid H. Ardakani, Elizabeth Watt, Yishu Song, Andrew Kingston, Jason M. E. Ahad

**Affiliations:** 1 Natural Resources Canada, Geological Survey of Canada, Calgary, AB T2L2A7 Canada; 2 ConocoPhillips Canada, Calgary, AB T2P3H7 Canada; 3 ConocoPhillips, Houston, TX 77079 USA; 4 Natural Resources Canada, Geological Survey of Canada, Québec, QC G1K 9A9 Canada

**Keywords:** Biogeochemistry, Biogeochemistry, Natural hazards, Ocean sciences, Energy, Organic chemistry, Fossil fuels

## Abstract

Some of the parental material for hydrocarbons produced from low-permeability reservoirs in Western Canada corresponds to thermal products from biodegraded oil. This has been proved by the occurrence of framboidal pyrite, which is often formed during microbial sulfate reduction (MSR). In addition, the identified pyrite framboids are associated with the presence of phosphorus (P). Phosphorus (as phosphate) is a key nutrient and energy carrier for sulfate-reducing bacteria. The pyrite-P assemblage occurs embedded in solid bitumen (thermal residue), which confirms that migrated hydrocarbons provided the environment for microbial growth. Molecular products of severe biodegradation such as 17-nortricyclic terpanes were also detected. Biodegradation effects have been masked not only by thermal degradation of biodegraded oil during maximum burial, but also due to hydrocarbon mixing with late gas-condensate charges. Suitable conditions for biodegradation (< 80 °C, basin uplift) occurred during the Early Cretaceous. The confirmation of paleo-biodegradation means that there was a significant hydrocarbon loss that we have not accounted for. Likewise, MSR and Early Cretaceous seawater sulfate might have played an important role in the generation of the hydrogen sulfide (H_2_S) detected today.

## Introduction

In recent years, petroleum exploration and production activities have placed major attention on hydrocarbon fluids with lower carbon footprint compared to crude oil. These fluids typically correspond to the gas-condensate range (c.a. C_1_–C_18_ carbon number) and are produced unconventionally worldwide. Unconventional low-permeability hydrocarbon reservoirs are challenging to assess, geochemically, particularly when they exhibit hybrid characteristics. The “hybrid’’ term has been used rather ambiguously, but we will refer to the definition by Jiang et al.^1^ to indicate reservoirs that having variable porosity and permeability (shale intervals to siltstone and sandstone) require hydraulic fracturing to produce hydrocarbons. They contain petroleum that can originate not only from primary in-situ organic matter but also from the thermal degradation of migrated/trapped hydrocarbons. An example can be found in the Western Canadian Sedimentary Basin (WCSB), specifically the Lower Triassic Montney Formation, which contains vast petroleum accumulations that resulted from thermal degradation of migrated hydrocarbons as this geological unit reached maximum burial and temperature^2–5^. Original organic matter has also been recognized in the Montney Formation from Alberta^6^, but its contribution to the total hydrocarbon production remains unknown.

Up until now, the sources of hydrocarbons that migrated into Montney reservoirs are poorly constrained. Some correlation has been established with the Middle Triassic Doig Formation and the Lower Jurassic Gordondale Member of the Fernie Formation^5,7,8^. However, many other Montney fluids lack similarity with any known Triassic and/or older source rocks of the WCSB^7^.

Clear challenges in the discrimination of hydrocarbon origin within Montney are due to the thermal degradation of migrated hydrocarbons, which overprints source signatures. Other mechanisms that could mask original geochemical hydrocarbon properties include alteration via biodegradation^9^. The possibility of biodegradation of Montney fluids has not been touched upon due to the lack of clear evidence in currently produced hydrocarbons (e.g., oils and condensates with regular *n*-alkane patterns). However, several publications have reported framboidal pyrite as a product of microbial activity within Montney tight intervals^8,10–12^ with no concrete explanation of how the same microbes could have altered the migrated oil.

The redistribution of hydrocarbons can also mask alteration processes such as biodegradation. Although target intervals of the Montney Formation today represent a tight rock that requires stimulation for hydrocarbon production, they preserve signatures of multiple stages and mechanisms for hydrocarbon migration that are yet complex to understand^2,13^. These fluid dynamics are important not only for the purpose of petroleum system analysis, but also to address key production issues such as the presence of hydrogen sulfide (H_2_S) within the formation.

**Figure 1:**
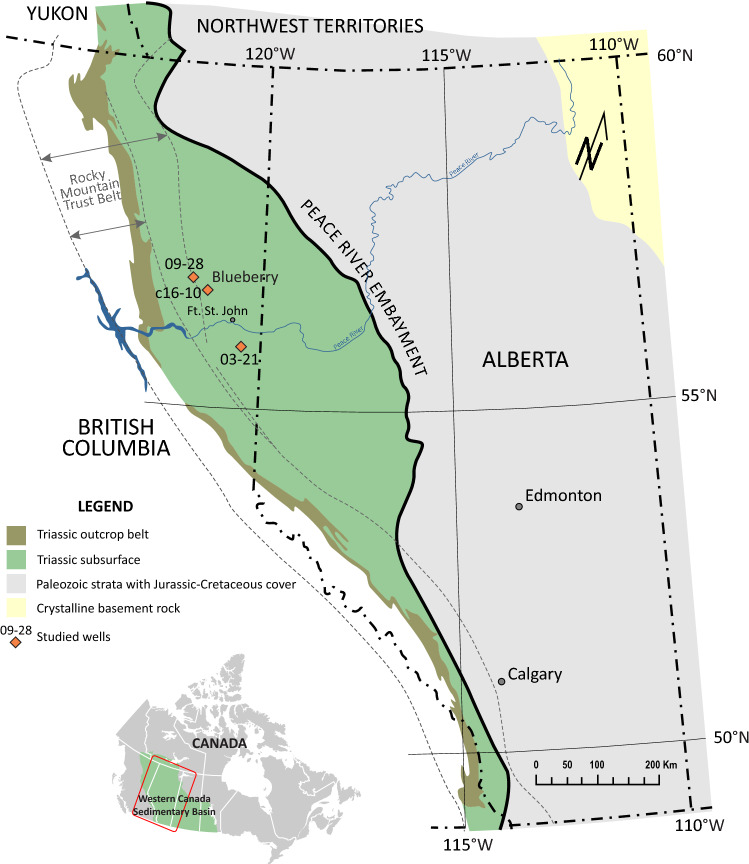
Location of the study well C16-10 within the framework of the Montney Formation. Blueberry area and wells 09–28 and 03–21 are shown as reference for the basin models discussed later in the manuscript. Base map modified after Furlong et al.^14^, CC BY open access license https://creativecommons.org/licenses/by/4.0/.

To address the abovementioned concerns, we have combined organic petrography, scanning electron microscopy/energy dispersive spectroscopy (SEM/EDS), secondary ion mass spectrometry (SIMS), and molecular and isotope geochemistry of core samples corresponding to an interval of the Lower Triassic Montney Formation from British Columbia, Canada (Figs. 1, S1). These geochemical analyses will allow us to find answers for the following aspects: (i) evidence of biodegradation of migrated hydrocarbons prior to Montney maximum burial and (ii) indicators of hydrocarbon mixing as a result of migration.

## Results

**Figure 2:**
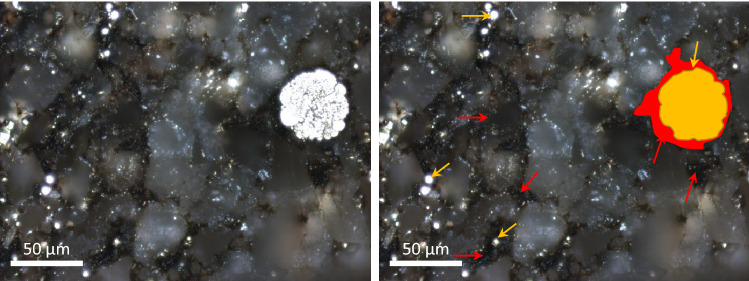
Optical identification of pyrite. Original microphotography on the left panel. The right panel indicates solid bitumen (red arrows) and framboidal pyrite (yellow arrow) in sample X12149, with an example of pyrite framboids (yellow) in solid bitumen (red).

**Figure 3:**
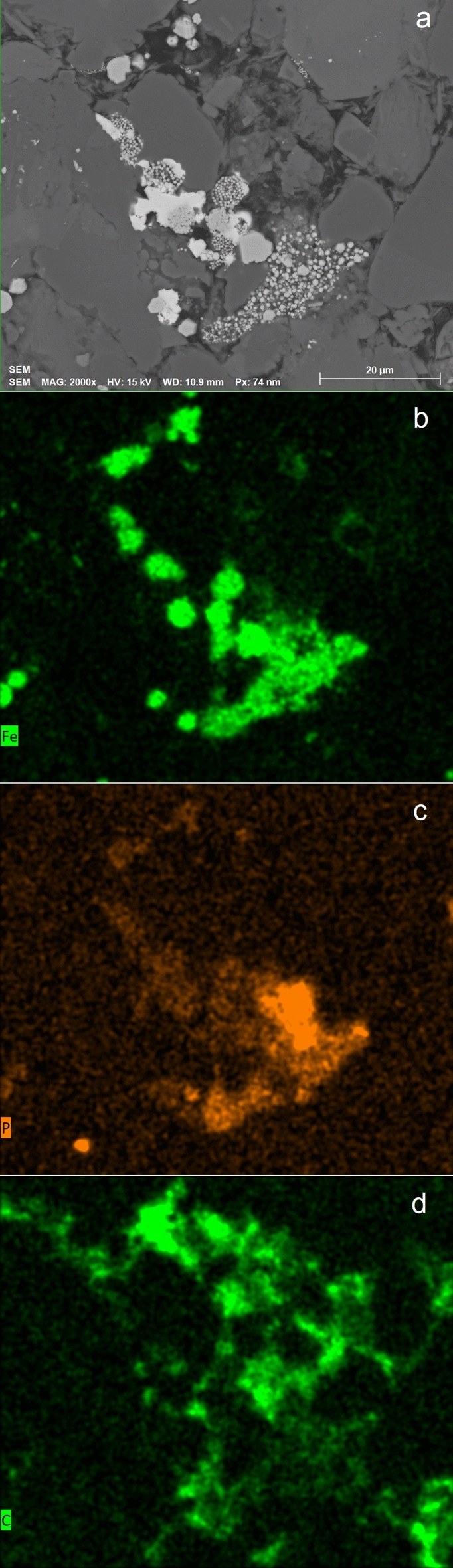
An SEM-EDXS example from sample X12149 (**a**) showing the presence of (**b**) iron (Fe), (**c**) phosphorous (P), and (**d**) carbon (C).

The organic petrography analysis provides evidence for the ubiquitous presence of framboidal pyrite (raspberry-shaped spherical aggregates; FeS_2_) in all samples (Fig. 2). Abundant framboidal pyrite, formed in intergranular pore spaces in close association with pore-filling solid bitumen, suggests their formation after hydrocarbon emplacement^11^. Framboidal pyrite has been previously attributed to microbial activity because of the morphological similarity with bacterial and nano-bacterial colonies that reduce sulfate^15,16^, which subsequently reacts with iron oxides in pore water^17–19^. The morphology of bacterial colonies is preserved during mineral uptake to form the framboids^20^. Another compositional characteristic important to highlight is the presence of phosphorus (P) associated with the pyrite framboids (Fig. 3a–c) and organic carbon (Fig. 3d). Phosphate is an essential nutrient and a component of lipid membranes, which carries the energy that is used to activate sulfate during MSR^21,22^.

**Figure 4:**
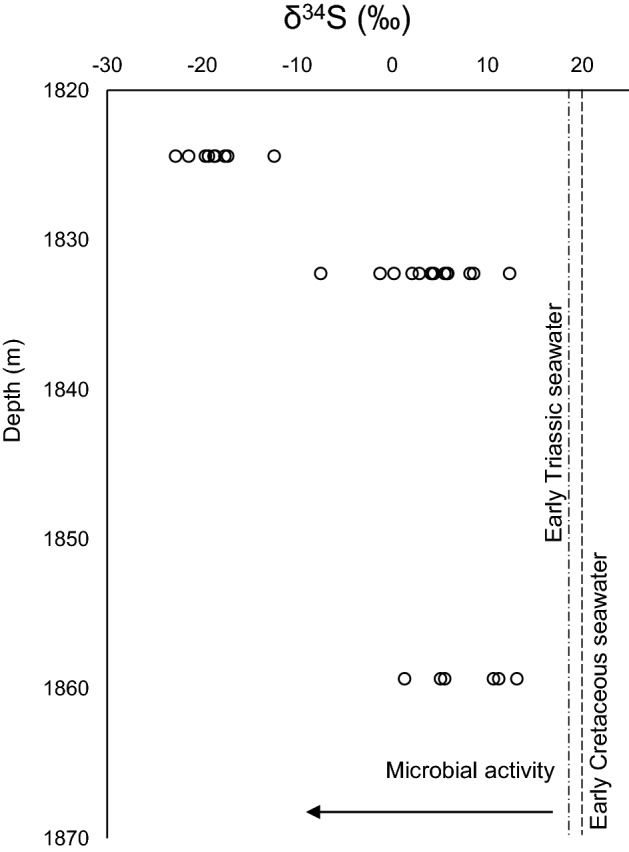
The δ^34^S values of samples from the study core, compared with the δ^34^S values of Early Triassic seawater^22^ and Early Cretaceous seawater^23^.

To further assess the origin of pyrite, SIMS results are shown in Fig. 4, and indicate δ^34^S values of pyrite ranging from − 23 to 12 ‰. In addition, the δ^34^S values become more positive with increasing depth, and are lower than the δ^34^S values of Early Triassic (Montney deposition time) and Early Cretaceous (potential time of biodegradation event) seawater^23,24^.

**Figure 5:**
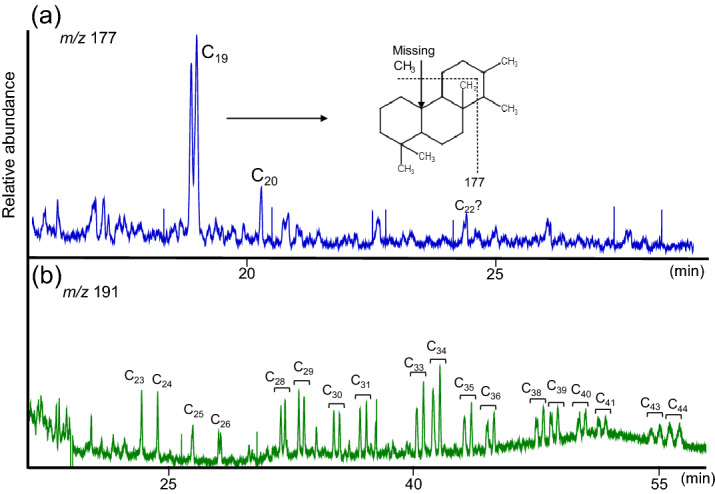
Molecular traces from the saturate fraction of sample X12149 as shown in (**a**) the m/z 177 and (**b**) m/z 191 fragmentograms. Labels in panel b indicate the number of carbon atoms.

As well, molecular characteristics of the samples were assessed for signatures of biodegradation. We may anticipate that thermal degradation has decreased the abundance of typical biomarkers for geochemical assessment (due to thermal cracking). For example, hopanes and steranes were below detection limit in all rock extracts. Nonetheless, we identified the presence of two peaks in the m/z 177 fragmentogram of the saturate fractions (Fig. 5a). Generally, the m/z 177 trace is used to identify 25-norhopanes, which are commonly associated with the process of biodegradation^25,26^. The 25-norhopanes form due to the loss of a methyl group (carbon number 25) during microbial degradation of hydrocarbons^27,28^, or simply from original organic matter, but remain stable because of greater resistance to biodegradation compared to other biomarkers^29^. The samples do not show 25-norhopanes or any other nor-hopanes series (e.g., 28-nor and 25, 28-dinorhopanes) in Fig. 5b. After comparison with previously reported mass spectra^30,31^, the two peaks identified in the m/z 177 trace correspond to the de-methylated epimers (S, R) of C_19_ tricyclic terpane (17-nortricyclic terpanes) as illustrated in Fig. 5a (see also Fig. S2). The C_20_ de-methylated homologue is tentatively assigned based on an extra methyl group on the mass spectrum. Minor peaks on the m/z 177 fragmentogram probably correspond to other de-methylated homologues, but the abundance is low for accurate identification.

Additionally, the fragmentogram of m/z 191 proved the presence of extended tricyclic terpanes (Fig. 5b). The origin of these compounds is unknown, as reported in previous studies^9,32,33^. Nonetheless, it is clear that these compounds are highly resistant to biodegradation as found by Huang et al.^34^. The same authors observed that extended tricyclic terpanes remain after other compound classes, such as hopanes and steranes, have been removed via microbial activity. All the samples analyzed in this study contain extended tricyclic terpanes (Fig. 5b) and 17-nortricyclic terpanes.

As mentioned in the Introduction, alteration processes, such as biodegradation and thermal degradation, can also be difficult to characterize because of hydrocarbon mixing. Clear evidence of petroleum mixtures was found in the petrographic results as described below.

**Figure 6:**
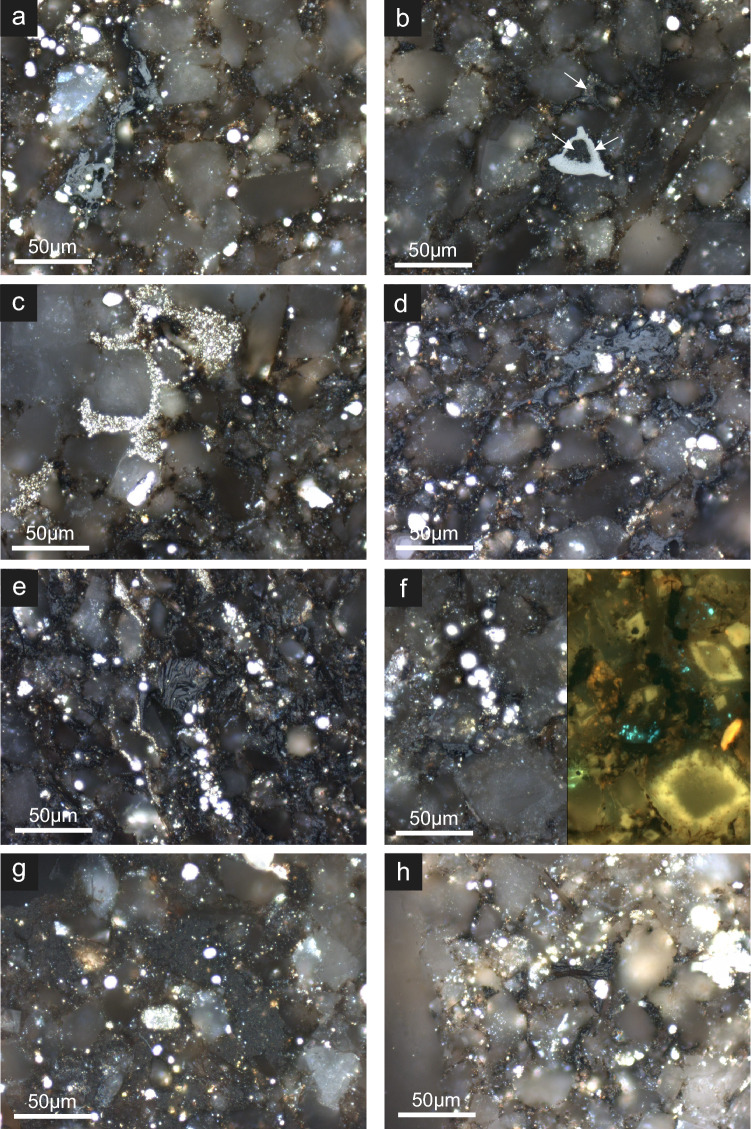
Photomicrographs of Montney samples with incident white light under oil immersion with × 50 objective unless specified otherwise. (**a**) Light gray pore-filling solid bitumen along with small framboidal pyrite. Note the close association of bitumen with framboidal pyrite. (**b**) Inertinite (combusted plant particle) in a siltstone matrix, the central hole in inertinite filled with solid bitumen. Fine-crystalline pyrite started to form on pore-filling solid bitumen (white arrow). (**c**) The large part of intergranular pores stained with hydrocarbons covered by late fine-crystalline pyrite. (**d**) Pore-filling solid bitumen occluded intergranular pores in a coarse-grained siltstone matrix. (**e**) Pore-filling solid bitumen associated with small framboidal pyrite. Pores between mica/clay platelets filled with solid bitumen. (**f**) Pair view under white incident (left) and right ultraviolet (UV) light (right) of pore-filling solid bitumen and blue fluorescing hydrocarbon-bearing fluid inclusions within dolomite cement suggest evidence of hydrocarbon migration. (**g**) Large pores filled with solid bitumen. Note the darker gray shade of solid bitumen and its granular texture. The solid bitumen has lower %BRo value in comparison to dominant higher %BRo solid bitumen phase. (**h**) Pore-filling solid bitumen associated with fine framboidal pyrite.

The predominant organic matter maceral in all studied samples is pore-filling solid bitumen (Fig. 6) and rare inertinite (Fig. 6B). In addition to solid bitumen, the samples commonly contain intergranular pores and grain contacts stained with hydrocarbon (Fig. 6A–C). Two populations of solid bitumen with distinct random bitumen reflectance (BRo) were identified: (1) pore-filling dark gray highly granular (Fig. 6B,C,G) mean BRo of 0.36 ± 0.07% (VRo_eqv_ 0.41%) and (2) predominantly pore-filling and intercrystalline-filling solid bitumen (Fig. 6A,D,E–G) with smooth surface and higher BRo (mean = 0.87 ± 0.09%; VRo_eqv_ 0.94%). In some samples, solid bitumen filled the pore spaces between clay platelets (Fig. 6G,H). At this stage, the presence of two prominent bitumen types with distinct %BRo values suggest mixing of at least two hydrocarbon charges of different thermal nature, discussed further in the next section.

Assessing hydrocarbon charges from the molecular geochemistry perspective was more challenging because traditional saturate biomarkers were below detection limit (e.g., steranes and hopanes). Typical thermal maturity parameters using alkylphenanthrenes and alkylnaphthalenes were found to be either constant or equilibrated (see Table S1 and Fig. S3). Therefore, the variability observed in different molecular indices calculated in this study is not explained by significant vertical thermal maturity variations (perhaps not expected for this short 40 m interval).

Considering alternative molecular tools, we compared the abundance of polycyclic aromatic hydrocarbons (PAHs) to 1,3,6,7-tetramethylnaphthalene (1,3,6,7-TeMN) which is considered to be of prominent marine origin^35–37^. The same ratio has successfully been applied in several studies to evaluate the marine versus terrestrial character of organic matter^35,38^. In our case, we cannot strictly apply the same concept because there is no significant terrestrial organic matter contribution to the hydrocarbon accumulations within the Montney Formation^6,8,10,39^. For example, although some xenospheres were observed (combusted terrigenous organic matter, Figs. 6B and S4), PAHs often associated with combustion of land plants such as pyrene, fluoranthene, benzofluoranthenes, coronene and benzoanthracene, were not detected. Molecular analysis detected chrysene, methylchrysenes, perylene and methylperylenes (Fig. S5), which could be combustion products of land plants, but are also found in marine-derived hydrocarbons^40–43^.

**Figure 7:**
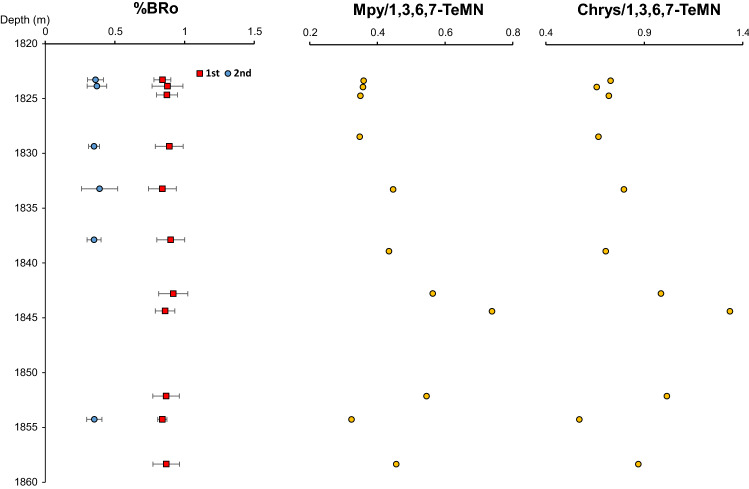
Depth variability of methylpyrenes (Mpy) and chrysene (as a ratio to 1,3,6,7-trimethylnaphthalene) compared with the identification of one or two types of solid bitumen. Smooth surface bitumen (red), granular bitumen (blue).

Nonetheless, under the premise that PAHs are thermally more stable than other aromatic and aliphatic compound structures, we have used PAH/1,3,6,7-TeMN ratios as indicators of thermal alteration/maturation (of the biodegraded oil). For example, we calculated the (4 + 1)-methylpyrenes / 1,3,6,7-TeMN and chrysene/1,3,6,7-TeMN ratios using peak areas from the m/z 216, 228 and 184 for methylpyrenes, chrysene and 1,3,6,7-TeMN, respectively (Table S1). None of these ratios show an increasing trend with depth as it would be expected for increasing temperature in the same direction (Fig. 7). Instead MPy/1,3,6,7-TeMN values are higher in the middle of the interval (e.g., 0.7 in sample X12140, 1844.4 m depth) and lower values are recorded at the top and bottom of the sequence (e.g., 0.4 in sample X12139, 1823.3 m depth; and 0.3 in sample X12150, 1854.3 m depth). Likewise, the highest Chry/1,3,6,7-TeMN value occurs at 1844.4 m (1.3, sample X12140), and the same ratio decreases at the top and the bottom of the sequence (e.g., 0.7 in sample X12139 and 0.6 in sample X12150; Fig. 7).

Figure 7 also shows that the highest PAHs to1,3,6,7-TeMN ratios are observed where the most prominent bitumen type (smooth surface solid bitumen) has the highest bitumen reflectance values (VRo_eqv_ 0.94%). Thus, the areas with higher PAH content and only one bitumen type seem to be consistent with an unmixed (or less mixed) more mature hydrocarbon accumulation. This is consistent with a relative increase of shale versus siltstone from 15:85 to 30:70 in the 1845–1855 m section of the core (Fig. S1) which limited the emplacement of subsequent gas-condensate charges.

**Figure 8:**
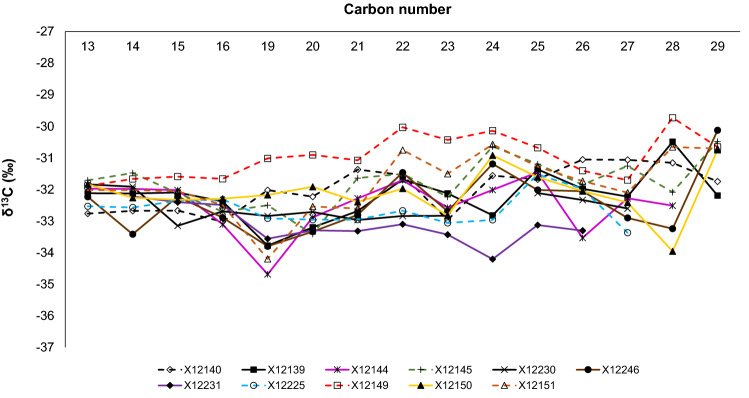
Stable carbon isotope profile of *n*-alkanes from the extracted samples. Dashed lines correspond to samples with one prominent bitumen type.

To further explore the possibility of mixing, Fig. 8 compares the stable carbon isotope profile of *n*-alkanes of the samples. In general, the δ^13^C values tend to overlap between − 34 and − 30 ‰ (V-PDB). In some samples, like X12140, the δ^13^C values tend to increase with carbon number, similar to profiles of hydrocarbon residues of thermally altered liquid petroleum^44–46^. Meanwhile, samples like X12145 have a δ^13^C flat to decreasing pattern between C_13_ and C_19_ and increasing pattern subsequently. Flat to decreasing δ^13^C values with carbon number are common in unaltered liquid hydrocarbons^44,47,48^. The semi v-shaped pattern of certain samples may be consistent with mixing of hydrocarbon charges of different origins. Other features that stand out include slightly higher values in the profile of sample X12149 (one bitumen type, VRo_eqv_ 0.94%), and more homogenous δ^13^C values in the C_13_-C_16_ range of all the samples.

## Discussion

For the first time, robust evidence is presented for hydrocarbon biodegradation within the Montney Formation as we have identified signals of microbial activity in the pore-filling bitumen. Subsequent to hydrocarbon emplacement, MSR facilitated the precipitation of framboidal pyrite (Figs. 2 and 6) after providing sulfide species that could react with iron available from clays and iron oxides^17,18^. The presence of P around the framboid aggregates (Fig. 3c) further confirms microbial activity since P is a nutrient and energy carrier for sulfate reducing bacteria (SRB)^22^.

It is important to mention that early diagenetic pyrite can also exhibit framboidal morphology associated to sedimentary organic matter in shales (including phosphate rich units)^49^. However, such possibility would be rare in our samples because (i) there is no evidence of primary organic matter in the study Montney section and (ii) the petrography indicated that hydrocarbon emplacement occurred first and the pyrite framboids formed later within the pore-filling hydrocarbons. The occurrence of framboidal pyrite embedded in bitumen has been reported in other Montney sections as well^8,12^ (Fig. S6). A similar process for framboidal pyrite formation has been reported in the biodegradation of hydrocarbon seeps^50^ and bacterial uranium mineralization^51^.

The microbial origin of pyrite was further confirmed by SIMS analysis. The sulfur isotope (δ^34^S) values of pyrite are within the isotope fractionation rate caused by MSR (15–75 ‰,^52,53^), assuming sulfur being sourced by Triassic seawater (18‰^22^) or Early Cretaceous seawater (20‰^23^), Early Cretaceous being the time for biodegradation as explained later in this section. The δ^34^S values of pyrite become more positive with depth probably due to recrystallization at higher temperatures (Fig. S7) and other processes such as thermochemical sulfate reduction (TSR) taking place, which involves sulfur isotope exchange with organics and H_2_S^12^.

The presence of de-methylated tricyclic terpanes and extended tricyclic terpanes (Fig. 3) suggests the microbial degradation of organic components as well. The presence of 17-nortricyclic terpanes is often associated with extreme levels of biodegradation^34,51^ although they could also represent thermal break-down products of larger biodegraded molecules such as 25-norhopanes. It remains unknown whether a specific biodegradation mechanism favored the C_19_ homologue, or its preferential occurrence is simply due to its thermal stability. In addition, extended tricyclic terpanes have not been removed by biodegradation due to their higher resistance to this process compared to regular hopanes and steranes. Other molecular signs of hydrocarbon biodegradation were probably erased due to thermal alteration caused by maximum burial and increasing temperature of the Montney Formation during the Late Cretaceous. Increasing temperatures might have also been responsible for the release of regular tricyclic terpanes (C_23_-C_26_, Fig. 5b) that were initially occluded or bounded to more complex polar structures and thus protected from the microbial degradation process^34,51^.

**Figure 9:**
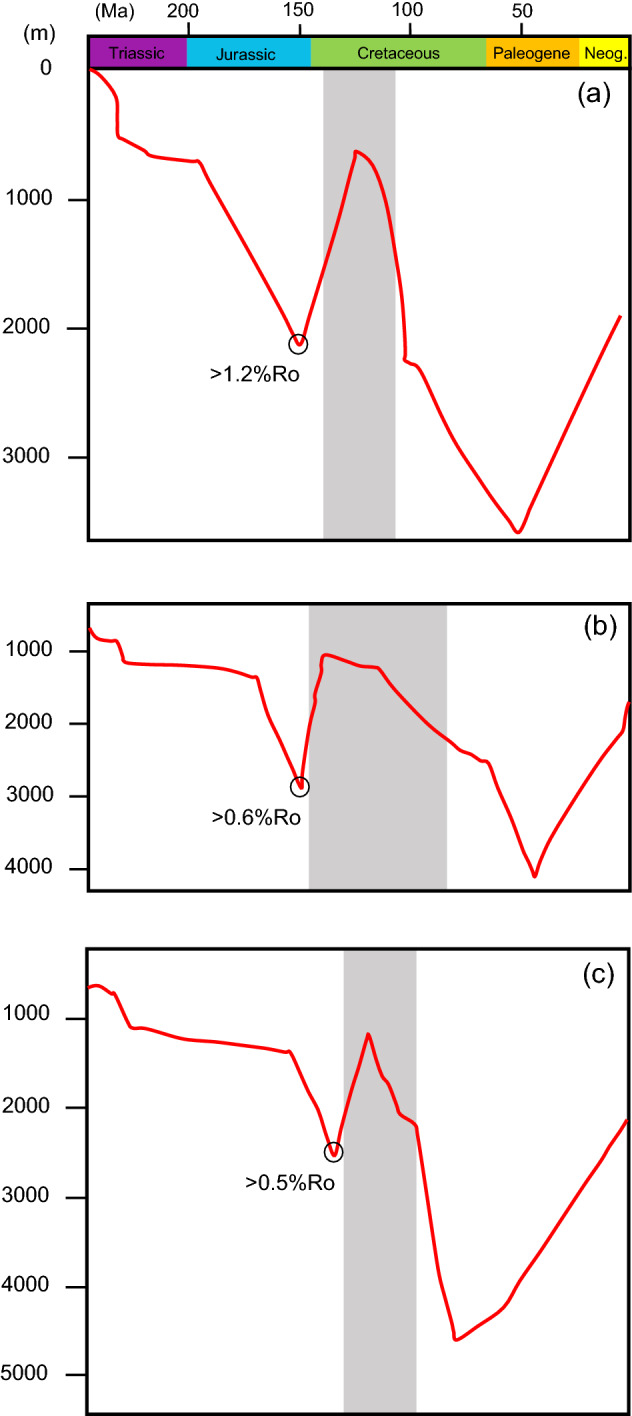
Burial history of the Montney Formation in British Columbia modified after (**a**) Watt et al.^54^ (well 09–28 in Fig. 1), (**b**) Mort^55^ (Blueberry field in Fig. 1), and (**c**) Ducros et al.^3^ (well 03–21 in Fig. 1). Gray bars represent approximate time with temperatures below 80 °C. Temperature and %Ro interpretations are from the same studies.

As implied above, our findings suggest that initial hydrocarbon charging, and biodegradation must have taken place prior to Montney’s maximum burial. Basin models^3,54,55^ speculate how the WCSB (northeast British Columbia side) experienced burial in the Late Jurassic (Fig. 9a–c), during which time Montney reached the oil window (Fig. 9a–c). According to Watt et al.^54^, the Middle Montney passed 1.2%Ro at the end of the Jurassic (Fig. 9a), whereas younger Triassic strata only approached to 0.7%Ro. This period of time was followed by uplift in the Early Cretaceous. We suggest that the uplift period was the time for the biodegradation to take place due to the accumulations being brought to shallower depth. Upward migration towards shallower intervals could have also been caused by depressurization during the Early Cretaceous uplift. Hydrocarbon migration could have started even during the Late Jurassic if suitable fluid volumes were available. It is important to mention that the models propose that Montney had temperatures below 80 °C during the Late Cretaceous uplift, which is a suitable temperature for microbial survival and activity^9^. Favorable temperatures for microbial activity could have lasted until Middle to Late Cretaceous times (Fig. 9b,c).

Based on the results of this study, we recommend applying similar molecular and mineralogical tools employed here to evaluate signatures of biodegradation in other Montney sections. Knowing the intra-formational extension of biodegradation is important as biodegraded oil represents a different parent material for an unknown portion of the hydrocarbons produced today. For example, some variability observed in produced fluid type might be attributed to their origin from thermal degradation of unaltered migrated oil versus biodegraded oil^56,57^. Furthermore, there is an unknown amount of hydrocarbon loss (via microbial degradation) that we have not accounted for.

Additional fluid migration occurred in the Eocene due to basin uplift^2,13,54^. Evidence of this subsequent hydrocarbon migration in the studied core is represented by the occurrence of two populations of solid bitumen (Fig. 7). One of these bitumen types corresponds to a higher thermal maturity (0.9%VRo_eqv_), which represents the original maximum thermal maturity reached by the studied section. Higher PAH/1,3,6,7-TeMN ratios associated with such bitumen may be a consequence of thermal maturation though 1,3,6,7-TeMN might have already been depleted because of biodegradation. We do not investigate the origin of this hydrocarbon charge. However, a recent study suggests that hydrocarbons could have migrated laterally from the west and emplaced within the studied Montney section as early as 190 Ma (Early Jurassic), and at temperatures of 40–50 °C^54^.

A second bitumen type with a thermal maturity of 0.4%VRo_eqv_ most likely resulted from the precipitation of insoluble compounds after hydrocarbon mixing with a subsequent light hydrocarbon charge. A low thermal maturity plus a highly granular texture also supports the bitumen precipitation hypothesis. The apparent maturity of 0.4%VRo_eqv_ is thus not indicating an early hydrocarbon generation process but rather represents a textural artifact. The molecular characteristics of the samples make evident that the later migrated charge consisted of lighter components since there is an increase of 1,3,6,7-TMN with respect to heavier aromatic structures (PAHs; Fig. 7). As well, light end *n*-alkanes have a slightly different carbon isotope profile compared with the C_19+_ compound range (Fig. 8). The gas-condensate must have migrated from deeper (more mature) intervals^13^. Migration of lighter components in the gasoline range has been reported as a common characteristic within Montney reservoirs^13^.

It is yet a question whether the carbon isotope profiles in Fig. 8 represent in any way the signature of the early biodegraded oil. However, since the biodegradation level is interpreted as severe, we may anticipate that the *n*-alkanes of the early migrated oil were fully removed. In addition, the bulk carbon isotope ratios of the saturate fractions vary within a narrow range (− 32.0 to − 31.4 ‰, see Table S3), which suggests that the isotope signature of the biodegradation was overprinted by thermal degradation during Montney’s maximum burial.

**Figure 10:**
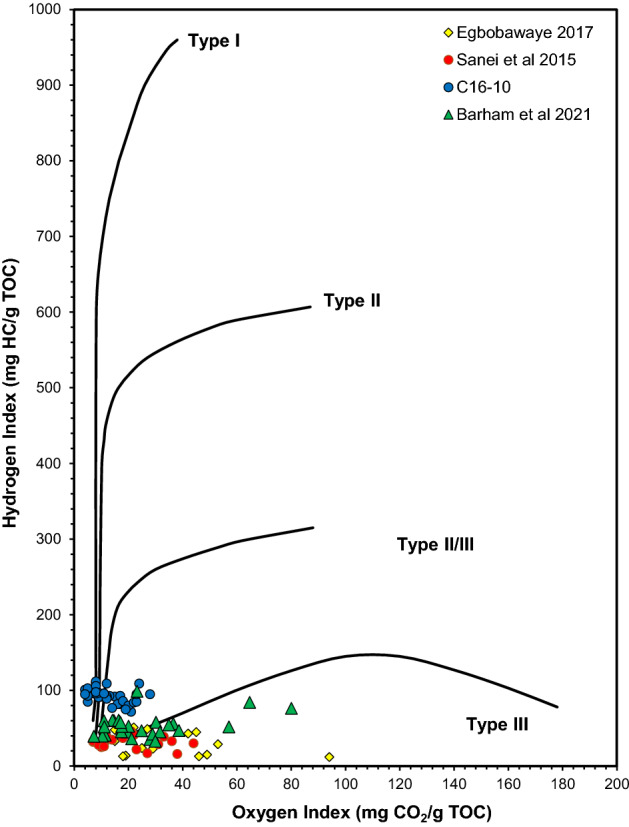
Pseudo-van Krevelen diagram showing previously assigned type III organic matter for the Montney Formation in British Columbia^8,57,58^ compared with the samples from this study (C16-10).

It is worth noting that if biodegraded hydrocarbons were the parental material for the produced fluids during maximum burial of Montney, the same material would be more gas-condensate prone given its compositional characteristics resembling kerogen type III/IV^57,58^. This leads us to a reinterpretation of previously reported pyrolysis data of Montney samples from British Columbia, which had indicated kerogen type III/IV though it is known (based on biomarkers and basin history) that the organic matter of all potential source rocks (including Montney) is prominently marine^6,7,39^. It is very likely that the parental organic material in those pyrolyzed samples was a biodegraded residue, which typically has higher oxygen content and more complex structures resembling kerogen type III/IV^57,58^. Some examples of these pyrolysis data are shown in Fig. 10^8,59,60^, including the samples from this study which also indicate an apparent type II/III organic matter input.

In brief, this research has made clear that the Montney’s hybrid low-permeability reservoir characteristics necessitates a redefined petroleum system analysis to account for: (i) hydrocarbon biodegradation and thermal degradation of biodegraded oil; and (ii) hydrocarbon mixing with potential precipitation of destabilized hydrocarbon components (including its effects on fluid quality and petro-physical properties such as porosity and permeability). The understanding of all of the above is critical to accurately interpret other molecular and isotope data for fluid to fluid and fluid to source correlations. Furthermore, the biodegradation process is important because hydrocarbons are degraded by sulfate reducers which generate H_2_S, and H_2_S can accumulate if limited metals are available to scavenge H_2_S for sulfide mineralization. This introduces the questions of H_2_S in Montney being associated (or not) to the Early Cretaceous biodegradation event and Early Cretaceous seawater sourcing sulfate (affecting current interpretations of S isotopes of H_2_S gas). Additional studies are needed to fully assess the regional extent of biodegradation in the Montney, which is essential to accurately evaluate the characteristics of hydrocarbons and hydrocarbon migration in the basin.

Although we did not attempt fluid-source correlations in this study, the same are encouraged for future research. We know, however, that condensates from British Columbia have shown mixed molecular distribution patterns, and carbon isotope profiles of *n*-alkanes with “v” shape^4^, similar to some of the extracts analyzed in this research.

## Methods

We performed a series of analyses on samples from a Montney Formation core in British Columbia, Canada (100/16-10-088-23W6/00; surface latitude 56.622, surface longitude -121.566). The approximately 40 m interval corresponds to the Upper Montney Formation consisting of a brown to dark brown quartzose siltstone with disseminated phosphate, white and black micro mica flakes and moderate carbonaceous. The siltstone is predominately cemented with silica and/or dolomite, whereas the matrix is mostly argillaceous^61^. Additional geological background for this core can be found in the supplementary material.

### Organic petrography

Organic petrography was carried out on 11 selected samples using polished blocks made with a cold-setting epoxy–resin mixture. The resulting sample pellets were ground and polished, in final preparation for microscopy, using an incident light Zeiss Axioimager II microscope system equipped with fluorescent light source and the Diskus-Fossil system for the reflectance measurements. Fluorescence microscopy of organic matter was carried out using ultraviolet (UV) G 365 nm excitation with a 420 nm barrier filter. Random bitumen reflectance (BRo) measurements were conducted under oil immersion (objective × 50) following ASTM (2014) standard. The standard reference for reflectance measurement was yttrium–aluminum-garnet with a standard reflectance of 0.906% under oil immersion.

### Scanning electron microscopy- energy dispersive X-ray spectroscopy

SEM–EDS was performed on mechanically polished organic petrography pellets. This work was conducted using the FEI Quanta 250 FESEM under low vacuum condition at the University of Calgary’s Instrumentation Facility for Analytical Electron Microscopy.

### Secondary ion mass spectrometry (SIMS)

Doubly polished thick Sects. (100 µm) were prepared from three depth (1824.4, 1832.2, and 1859.3 m) in the core. Following petrographic analysis, five regions of interest (ROI) containing representative pyrite grains were selected for SIMS analysis. ROIs were cored with a 2 mm drill bit and mounted in epoxy resin alongside a suite of internal pyrite reference materials. Detailed images of ROIs were collected with backscatter electrons via scanning electron microscopy for SIMS spot selection. Sulfur isotope ratios (^34^S/^32^S) were analyzed on an IMS-1280 multi-collector ion microprobe housed in the Canadian Centre for Isotopic Microanalysis at the University of Alberta. All sulfur isotope data is reported in standard per mil (‰) notation relative to Canyon Diablo Troilite (VCDT). Replicate analysis of reference materials was used to assess and correct for ^34^S^–^/^32^S^–^ instrumental mass fractionation and instrument drift during the analytical session. A ^34^S/^32^S value of 0.0441626 for VCDT (Ding et al., 2001) was used for measured δ^34^S normalization. Total uncertainty for pyrite δ^34^S measurements is ± 0.20 ‰ (2σ).

### Molecular analysis

A total of 11 rock samples were extracted for the molecular analysis of extracted bitumen using dichloromethane (DCM) in a soxhlet apparatus for 24 h. The bitumen extracts (10–40 mg) were separated into saturate, aromatic, resins and asphaltenes using column chromatography according to standard operation procedures of the organic geochemistry laboratory of the Geological Survey of Canada–Calgary. Saturate fractions were analyzed via gas chromatography with flame ionization detection (GC-FID, Fig. S8) and gas chromatography – mass spectrometry (GC–MS). Specific chromatographic details can be found in the supplementary material.

### Carbon isotope analysis of hydrocarbons

The saturate fractions were analyzed via gas chromatography – isotope ratio mass spectrometry (GC-irMS) to obtain compound-specific δ^13^C values. Chromatographic conditions were the same as those implemented for molecular analysis, and the specific details can be found in the supplementary material.

## Supplementary Information

Supplementary Information.

## Data Availability

The data discussed in this study can be found in the Supplementary Material.
