# Supplementary material for: Paleo-biodegradation and hydrocarbon mixing in a major hybrid petroleum reservoir
Source: Sci Rep. 2023 Mar 16;13:4357. doi: 10.1038/s41598-023-31350-z (PMC10020580; doi:10.1038/s41598-023-31350-z)
Supplement: Supplementary file 1 — Supplementary Information. [file 41598_2023_31350_MOESM1_ESM.docx]

**Paleo-biodegradation and hydrocarbon mixing in a major hybrid petroleum reservoir**

Jaime Cesar^a*^, Omid H. Ardakani^a^, Elizabeth Watt^b^, Yishu Song^c^, Andrew Kingston^a^, Jason M. E. Ahad^d^

^a^ Natural Resources Canada, Geological Survey of Canada, Calgary, AB T2L2A7, Canada.

^b^ ConocoPhillips Canada, Calgary, AB T2P3H7, Canada.

^c^ ConocoPhillips, Houston, TX 77079, United States

^d^ Natural Resources Canada, Geological Survey of Canada, Québec, QC G1K 9A9, Canada

* **Email:**  jaimerafael.cesarcol@ucalgary.ca

**Geological settings**

The Lower Triassic Montney Formation deposited in the northwestern margin of the North American craton during the Lower Triassic (Davies et al., 1997; 2018; Zonneveld and Moslow, 2018). Deposition of shoreface to offshore, and turbidite sediments took place on a clastic ramp along the northwestern margin of the Pangea Supercontinent in a collisional retro-foreland basin (Davies et al., 1997, 2018; Zonneveld and Moslow, 2018), integrating a mixed siliciclastic-carbonate depositional environment. Lithofacies include a complex succession of predominantly dolomitic siltstone and minor component of very fine- to fine-grained sandstone, with local bioclastic carbonate rocks (Davies et al., 1997, 2018; Zonneveld and Moslow, 2018).

The Montney Formation is unofficially divided into three members, Lower, Middle, and Upper members (Davies et al., 1997, 2018). The Lower Member corresponds to multi-cyclic very fine-grained sandstone and dolomitic siltstone. It overlies the Triassic unconformity on Permian and/or older strata, and it is overlain by the base of the Middle Member (where present) in the east, and by the Upper Member in the west. The Middle Member consists of a thick succession of bituminous dolomitic siltstone with interbedded very-fine grained sandstone. It overlies the Lower Member and is marked by a boundary of reworked clasts of the underlying units. The Upper Member corresponds to multicyclic, coarsening-up siltstones and very fine sandstones dominated by storm-related fabrics, with local dolomitized coquina facies; and it is overlain by the Doig Formation.

The samples analyzed in this study come from the core 100/16-10-088-23W6/00, located in British Columbia, Canada (surface latitude 56.622, surface longitude -121.566), which consists of the Upper Montney Formation. In the studied section, the formation corresponds to a brown to dark brown quartzose siltstone with disseminated phosphate, white and black micro mica flakes and moderate carbonaceous. The siltstone is predominately cemented with silica and dolomite, whereas the matrix is mostly argillaceous (McLeay Geological Consultants, 2006). Figure 1 includes some general characteristics of the core such as the Gamma API profile, measured porosity and permeability.

**
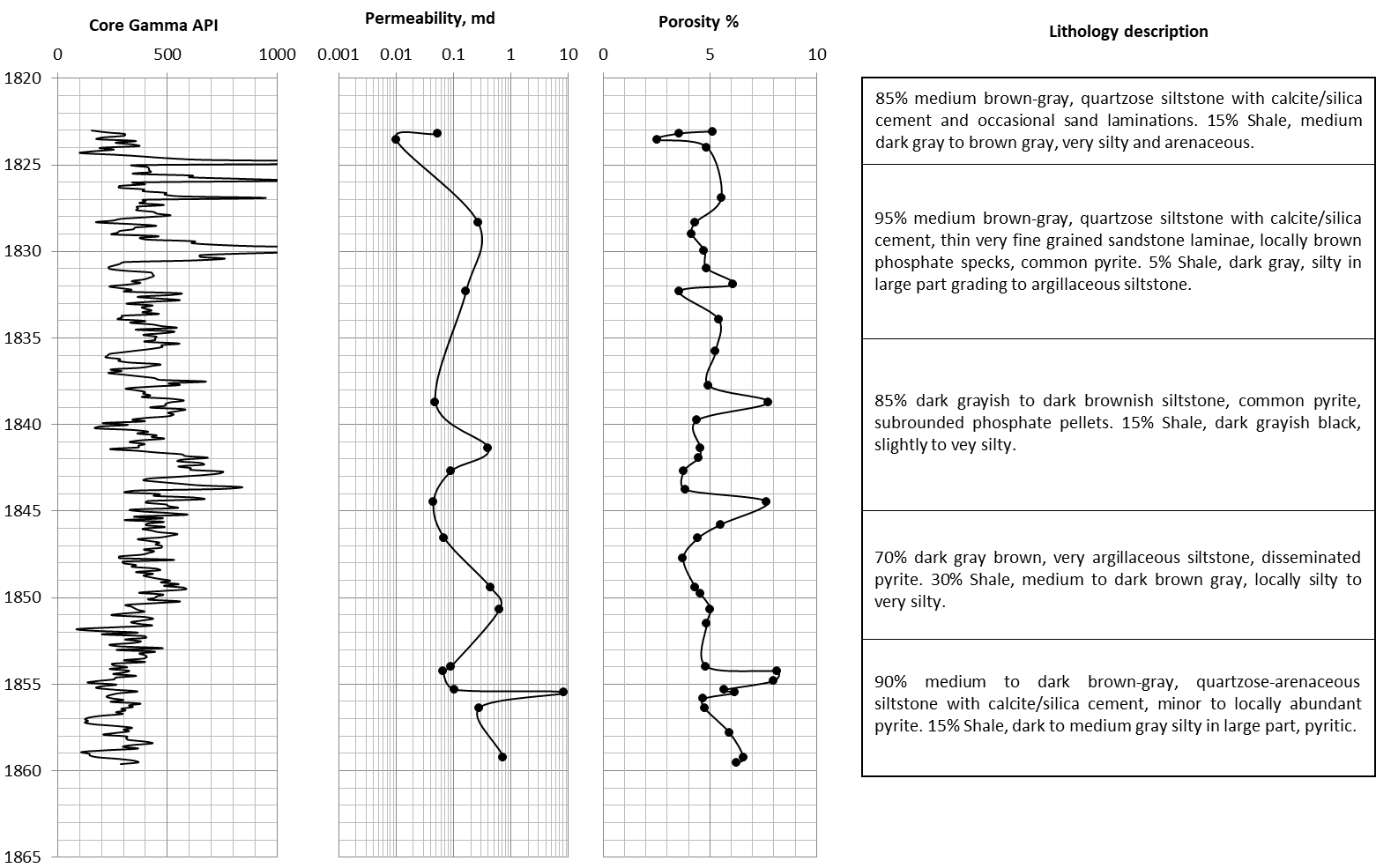
**

**Figure S1**. General characteristics of the core 100/16-10-088-23W6/00 including Gamma API profile, measured porosity and permeability, and descriptive lithology (McLeay Geological Consultants, 2006).

**Methods for molecular analysis**

A total of 11 rock samples were extracted for the molecular analysis of extracted bitumen using dichloromethane (DCM) in a soxhlet apparatus for 24 hours. The bitumen extracts were separated into saturate, aromatic, resins and asphaltenes using column chromatography according to standard operation procedures of the organic geochemistry laboratory of the Geological Survey of Canada – Calgary.

GC with flame ionization detection analysis of the saturated hydrocarbon fractions was completed on Agilent 7890B GC-FID. A 30 m × 0.25 mm × 0.25 μm DB-1 fused silica capillary column, with helium as carrier gas. The samples were injected using a split injector heated at 300 °C. The temperature program was set from 60 °C to 300 °C at a 6 °C/min ramp, and held at 300 °C for 30 min. The FID temperature was maintained at 300 °C.

Gas chromatography – mass spectrometry (GC–MS) analysis of saturated hydrocarbon fractions was carried out on an Agilent 7890B GC coupled to an Agilent 7000C Triple Quad MS operated in full scan mode. Split injection (2:1) was employed into a DB5msUI 30 m × 0.25 mm × 0.25 μm capillary column with helium as carrier gas at a flow rate of 1.2 mL/min. The initial temperature of the GC oven was 100 °C and then ramped to 320 °C at 5 °C/min (final temperature held for 15 min). Mass spectrometer ion source was operated at 70 eV ionization voltage.

Aromatic hydrocarbons were analyzed using an Agilent 6890 series GC coupled to a 5973 series Mass Selective Detector (MSD) operated in full scan mode. Splitless injection was employed into a HP-5ms phenyl methyl siloxane 30 m × 0.32 mm × 0.25 μm capillary column heated at 300 °C temperature. Helium was used as carrier gas at a flow rate of 1.3 mL/min. The GC oven temperature was programmed from 40 °C to 325 °C at a 4 °C/min ramp, and held at 325 °C for 15 min.

All molecular ratios calculated in this study are presented in **Table S1**.

**Table S1**. Molecular parameters measured on the rock extracts

| Lab ID | Pr/Ph | Pr/C17 | Ph/C18 | MPI | TMNr | TeMNr | Chrys | MPy | MPy/TeMN | Depth (m) |
| --- | --- | --- | --- | --- | --- | --- | --- | --- | --- | --- |
| X12139 | 1.50 | 0.81 | 0.67 | 0.61 | 0.91 | 0.91 | 0.73 | 2.60 | 0.36 | 1823.28 |
| X12144 | 1.48 | 0.84 | 0.65 | 0.60 | 0.90 | 0.89 | 0.66 | 2.37 | 0.36 | 1823.86 |
| X12145 | 1.49 | 0.77 | 0.63 | 0.58 | 0.90 | 0.89 | 0.72 | 2.74 | 0.35 | 1824.66 |
| X12230 | 1.41 | 0.88 | 0.68 | 0.59 | 0.88 | 0.86 | 0.67 | 2.74 | 0.35 | 1828.41 |
| X12146 | 1.50 | 0.86 | 0.69 | 0.62 | 0.91 | 0.90 | 0.80 | 2.54 | 0.45 | 1833.23 |
| X12231 | 1.57 | 0.78 | 0.46 | 0.59 | 0.90 | 0.90 | 0.70 | 2.68 | 0.43 | 1838.88 |
| X12225 | 1.50 | 0.76 | 0.50 | 0.61 | 0.90 | 0.90 | 0.98 | 2.81 | 0.56 | 1842.75 |
| X12140 | 1.43 | 0.85 | 0.70 | 0.63 | 0.88 | 0.89 | 1.33 | 2.59 | 0.74 | 1844.37 |
| X12149 | 1.53 | 0.86 | 0.67 | 0.63 | 0.91 | 0.90 | 1.01 | 2.42 | 0.55 | 1852.14 |
| X12150 | 1.31 | 1.02 | 0.80 | 0.66 | 0.91 | 0.90 | 0.57 | 2.47 | 0.32 | 1854.27 |
| X12151 | 1.50 | 0.86 | 0.68 | 0.62 | 0.90 | 0.91 | 0.87 | 2.50 | 0.46 | 1858.35 |

| MPI | [(3+2)-methylphenanthrene]/[phenanthrene+(9+1)-methylphenanthrene] | 178, 192 |
| --- | --- | --- |
| TMNr | 1,3,7-TMN/(1,3,7-TMN+1,2,5-TMN) | 170 |
| TeMN | 1,3,6,7-TeMN/[1,3,6,7-TeMN+(1,2,5,6+1,2,3,5)-TeMN] | 184 |
| Chrys | Chrysene/1,3,6,7-TeMN | 228, 184 |
| Mpy/ TeMN | (1+4 -methylpyrenes)/1,3,6,7-TeMN |  |
| MPy | 4-methylpyrene/1-methylpyrene | 216 |
| Pr/Ph | Pristane/phytane | GC-FID |
| Pr/C_17_ | pristane/n-C_17_ | GC-FID |
| Ph/C_18_ | phytane/n-C_18_ | GC-FID |

**Compound specific isotope analysis via gas chromatography – isotope ratio mass spectrometry (GC-irMS)**

The δ^13^C values of eight saturate fractions (X12139, X12140, X12144-X12146, X12149-X12151) were obtained using continuous flow GC-irMS technology. The setup consists of a Thermo Trace GC - GC-IsoLink system interfaced to a Thermo MAT 253 mass spectrometer via a Thermo Conflo IV. Aliquots of saturate fractions were manually injected into a helium carrier stream through the inlet of the GC using a gas tight syringe. Aliphatic hydrocarbons were separated on a GC DB-5 MS column (30 m × 250 μm with 0.25 μm of a bonded 5% phenyl–methyl–silicon stationary phase) before passing through a combustion reactor (maintained at 1000 °C). The temperature of the GC oven was programmed at 40 °C for 5 min, then ramped to 325 °C at 4 °C/min, and held isothermally for 15 min. Compound identification was achieved by comparisons of mass spectra and retention time with those available in the literature.

All hydrocarbon gas species were quantitatively converted to CO_2_ by passing the sample mixture through the combustion furnace. The separate CO_2_ gas pulses were then swept sequentially, by the He carrier gas, through a water trap (Nafion®) and into the open split interface, which facilitates steady continuous flow of the gas into the mass spectrometer. The δ^13^C values of the species were calculated by the instrument software (ISODAT 3.0), and are expressed in the usual delta (δ) notation in per mil (‰) relative to the international V-PDB standard. The samples were run in duplicates at a concentration of 2 mg/mL, and only the δ^13^C values with precision of ≤ 0.4‰ were reported. Every three sample measurements, a mixture of standards with known δ^13^C values was analyzed in order to ensure instrument accuracy. The carbon isotope ratio data obtained for peaks co-eluting, as well as those at very low signals, were not considered for interpretation.

Due to laboratory availability and time constraints, three other saturate fractions (X12225, X12230, X12231) were analyzed at the Delta-Lab of the Geological Survey of Canada (GSC-Québec) using similar conditions and instrumentation to those described above. All carbon isotope values are presented in **Table S2**.

**Table S2**. The δ^13^C values of the *n*-alkanes from the rock extracts (precision < 0.4 ‰)

| Sample | X12139 | X12144 | X12145 | X12230 | X12146 | X12231 | X12225 | X12140 | X12226 | X12149 | X12150 | X12151 |
| --- | --- | --- | --- | --- | --- | --- | --- | --- | --- | --- | --- | --- |
| δ^13^C_C13_ (‰) | -32.1 | -32.0 | -31.7 | -31.8 | -32.2 |  | -32.5 | -32.8 | -32.6 | -31.9 | -31.8 | -32.0 |
| δ^13^C_C14_ (‰) | -32.1 | -32.0 | -31.5 | -31.9 | -33.4 | -32.2 | -32.6 | -32.7 | -36.3 | -31.7 | -32.3 | -32.0 |
| δ^13^C_C15_ (‰) | -32.1 | -32.0 | -32.1 | -33.1 | -32.2 | -32.4 | -32.4 | -32.7 | -35.0 | -31.6 | -32.3 | -32.1 |
| δ^13^C_C16_ (‰) | -32.4 | -33.1 | -32.7 | -32.7 | -32.9 | -32.5 | -32.3 | -33.0 | -36.0 | -31.7 | -32.3 | -32.5 |
| δ^13^C_C19_ (‰) | -33.8 | -34.7 | -32.5 | -32.8 | -33.8 | -33.6 | -32.9 | -32.0 | -37.7 | -31.0 | -32.2 | -34.2 |
| δ^13^C_C20_ (‰) | -33.2 | -32.9 | -33.4 | -32.7 | -33.3 | -33.3 | -33.0 | -32.2 | -38.6 | -30.9 | -31.9 | -32.5 |
| δ^13^C_C21_ (‰) | -32.7 | -32.3 | -31.6 | -33.0 | -32.8 | -33.3 | -33.0 | -31.4 | -39.2 | -31.1 | -32.4 | -32.6 |
| δ^13^C_C22_ (‰) | -31.7 | -31.7 | -31.5 | -32.8 | -31.5 | -33.1 | -32.7 | -31.5 | -38.7 | -30.0 | -32.0 | -30.7 |
| δ^13^C_C23_ (‰) | -32.1 | -32.6 | -32.2 | -32.8 | -32.7 | -33.4 | -33.1 | -33.0 | -39.9 | -30.4 | -32.8 | -31.5 |
| δ^13^C_C24_ (‰) | -32.8 | -32.0 | -30.6 |  | -31.2 | -34.2 | -33.0 | -31.6 | -39.5 | -30.1 | -30.9 | -30.6 |
| δ^13^C_C25_ (‰) | -31.4 | -31.5 | -31.2 | -32.1 | -32.0 | -33.1 | -31.5 | -31.7 | -37.4 | -30.7 | -31.6 | -31.3 |
| δ^13^C_C26_ (‰) | -32.0 | -33.5 | -31.8 | -32.3 | -32.1 | -33.3 | -32.0 | -31.1 | -37.1 | -31.4 | -32.1 | -31.7 |
| δ^13^C_C27_ (‰) | -32.2 | -32.3 | -31.3 | -32.6 | -32.9 |  | -33.4 | -31.1 |  | -31.7 | -32.4 | -32.1 |
| δ^13^C_C28_ (‰) | -30.5 | -32.5 | -32.1 |  | -33.2 |  |  | -31.2 |  | -29.7 | -34.0 | -30.7 |
| δ^13^C_C29_ (‰) | -32.2 |  | -30.5 |  | -30.1 |  |  | -31.7 |  | -30.7 | -30.7 | -30.7 |

**Table S3**. The δ^13^C values of bulk saturate fractions

| **Sample** | **δ^13^C-_org_ (‰)** |
| --- | --- |
| X12139 | -31.4 |
| X12140 | -31.4 |
| X12144 | -31.6 |
| X12145 | -31.5 |
| X12146 | -32.0 |
| X12149 | -31.6 |
| X12150 | -31.7 |
| X12151 | -31.7 |
| X12225 | -31.8 |
| X12230 | -31.7 |
| X12231 | -31.8 |


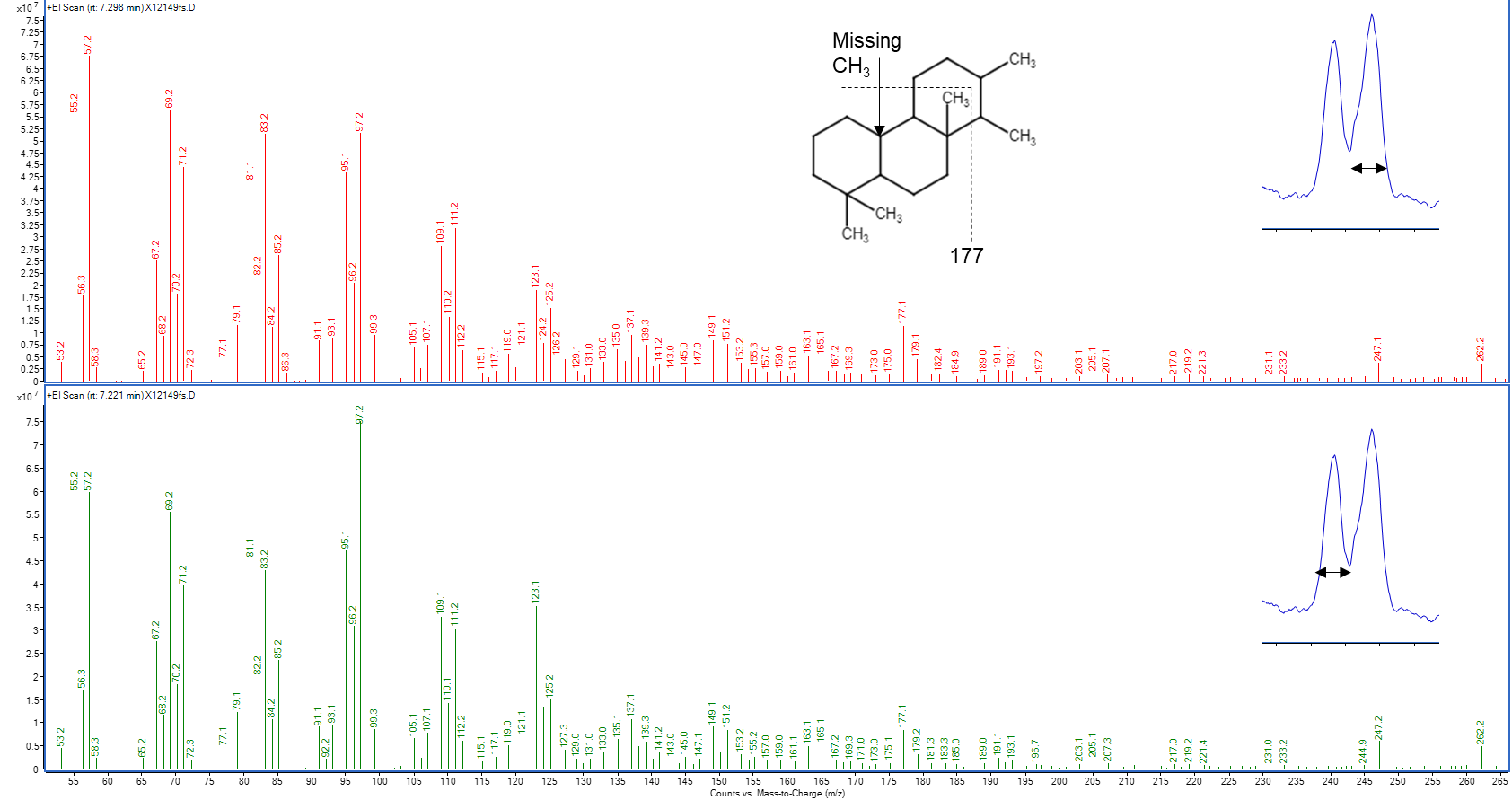


**Figure S2**. Mass spectra of the detected de-methylated tricyclic terpanes.


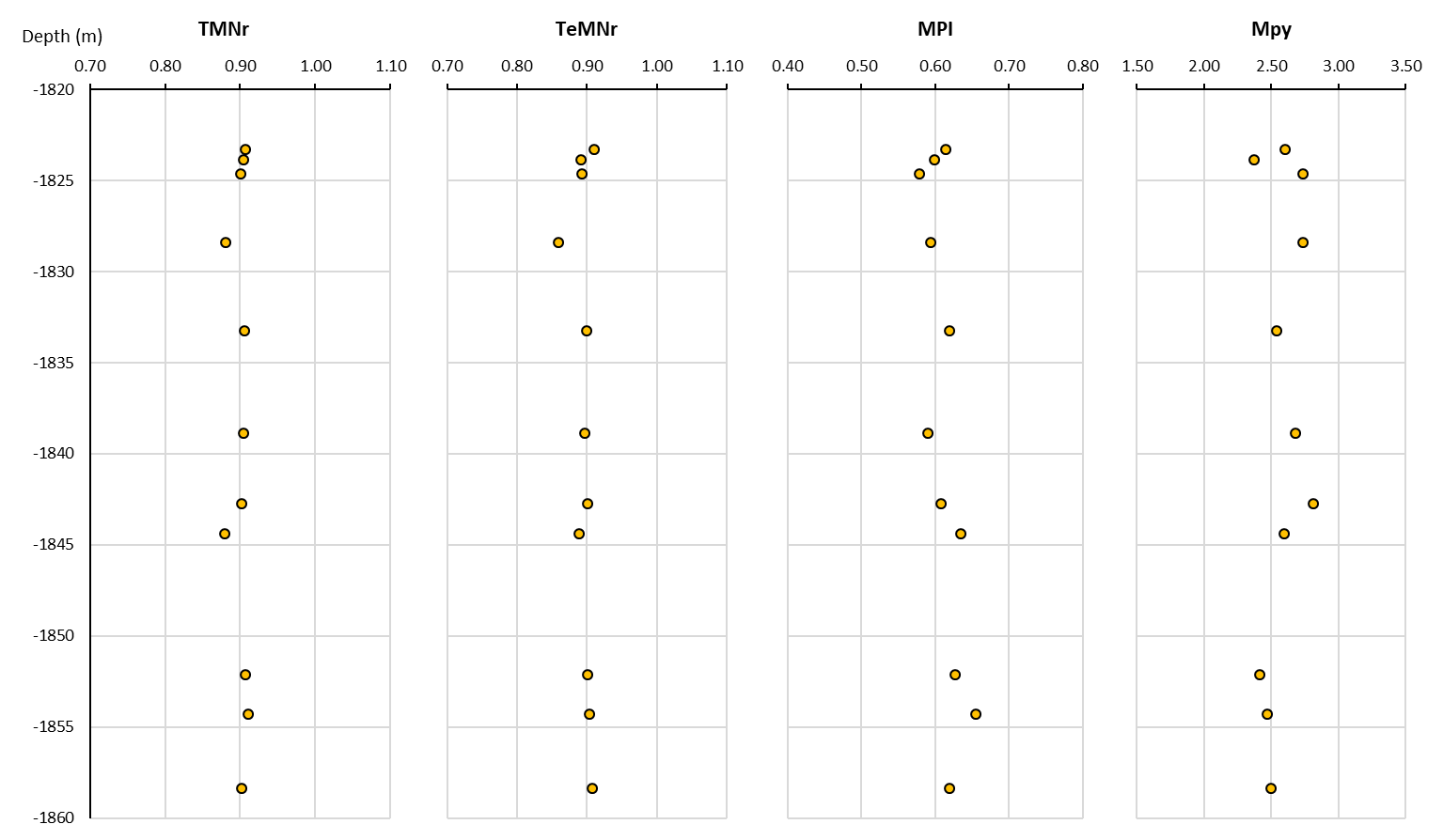


**Figure S3**. Depth profile of thermal maturity parameters including the TMN and TeMN ratios, the methyl-phenanthrene index (MPI) and the 4-methylpyrene / 1-methylpyrene ratio (Mpy). TMNr = 1,3,7-trimethylnaphthalene/(1,3,7-trimethylnaphthalene + 1,2,5-trimethylnaphthalene). TeMNr = 1,3,6,7-tetramethylnaphthalene/(1,3,6,7-tetramethylnaphthalene + 1,2,5,6-tetramethylnaphthalene + 1,2,3,5-tetramethylnaphthalene). MPI = (3+2 –methylphenanthrenes)/(1+9 –methylphenanthrenes + phenanthrene).


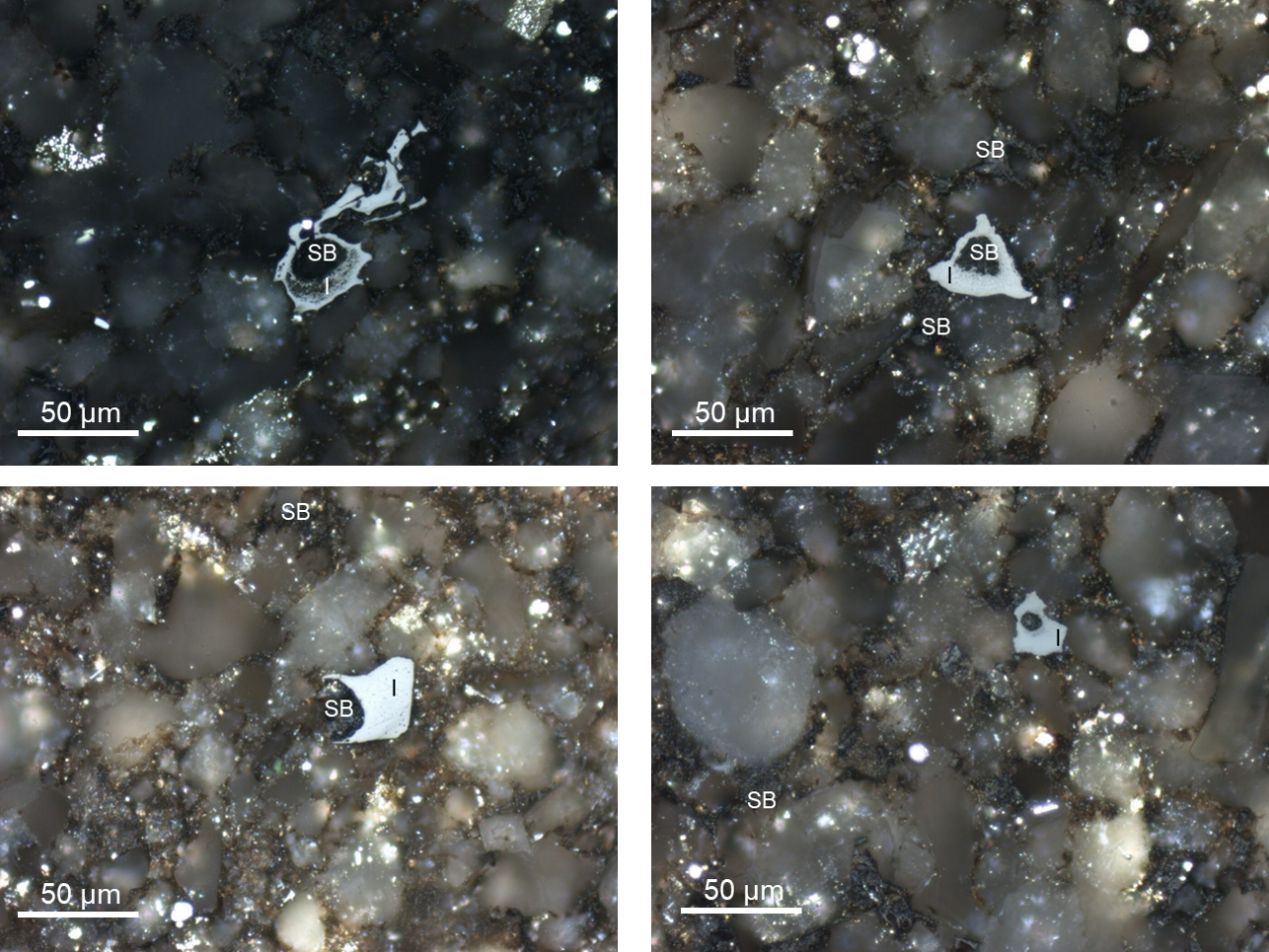


**Figure S4**. Petrographic examples of xenospheres (combusted terrigenous organic matter; denoted as I on the photos) and their association with solid bitumen (SB).


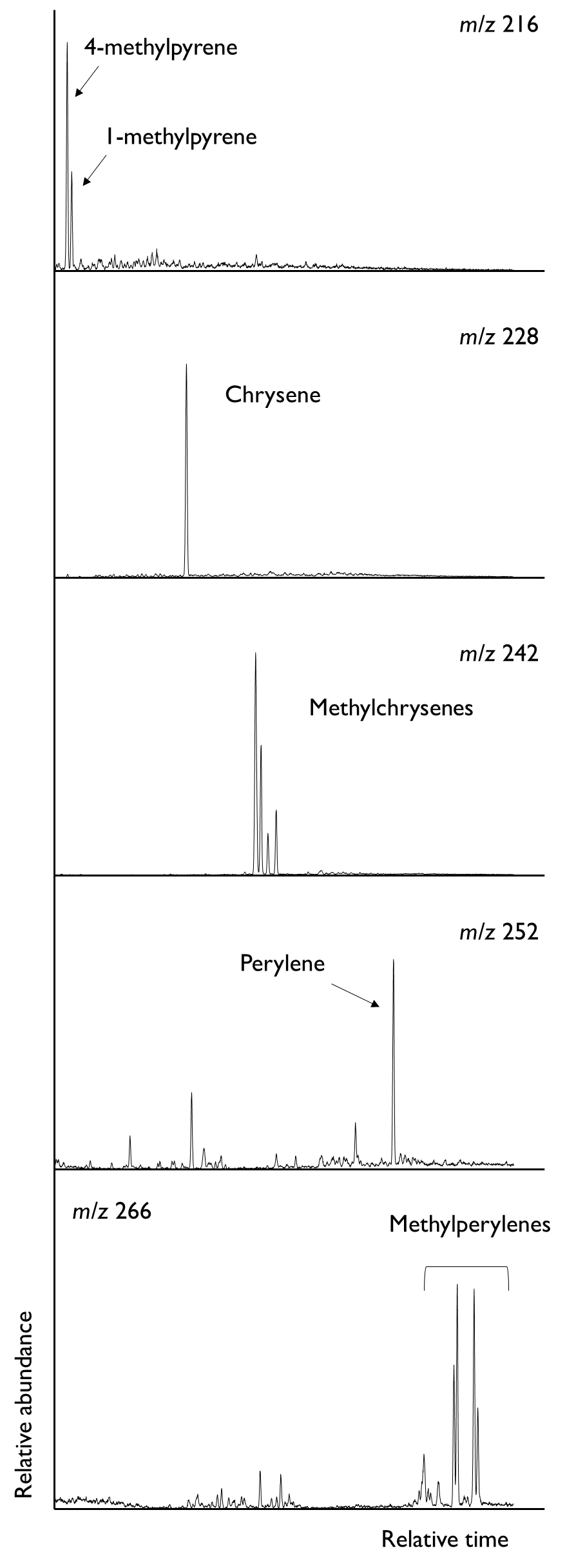


**Figure S5**. Polycyclic aromatic hydrocarbons detected in all the samples.

**Figure S6**. Framboidal pyrite occurrence associated with pore-filling bitumen in the well C-65-F/94-B-8 (UWI: 200C065F094B0800), British Columbia.


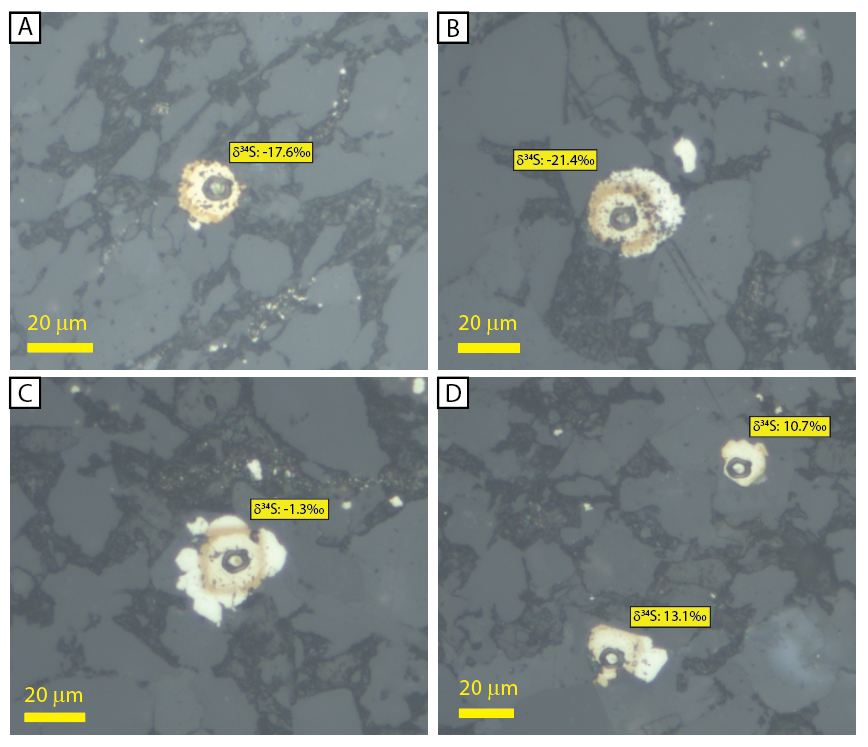


**Figure S7**: Examples of pyrite grains analyzed via secondary ion mass spectrometry. Framboidal pyrite (A,B) in the top of the core (1824.4 m). Subhedral to anhedral pyrite (C,D) is more common lower in the core (1832.2 m, 1859.3 m). Brownish circular marks and orifices were made during measurement.


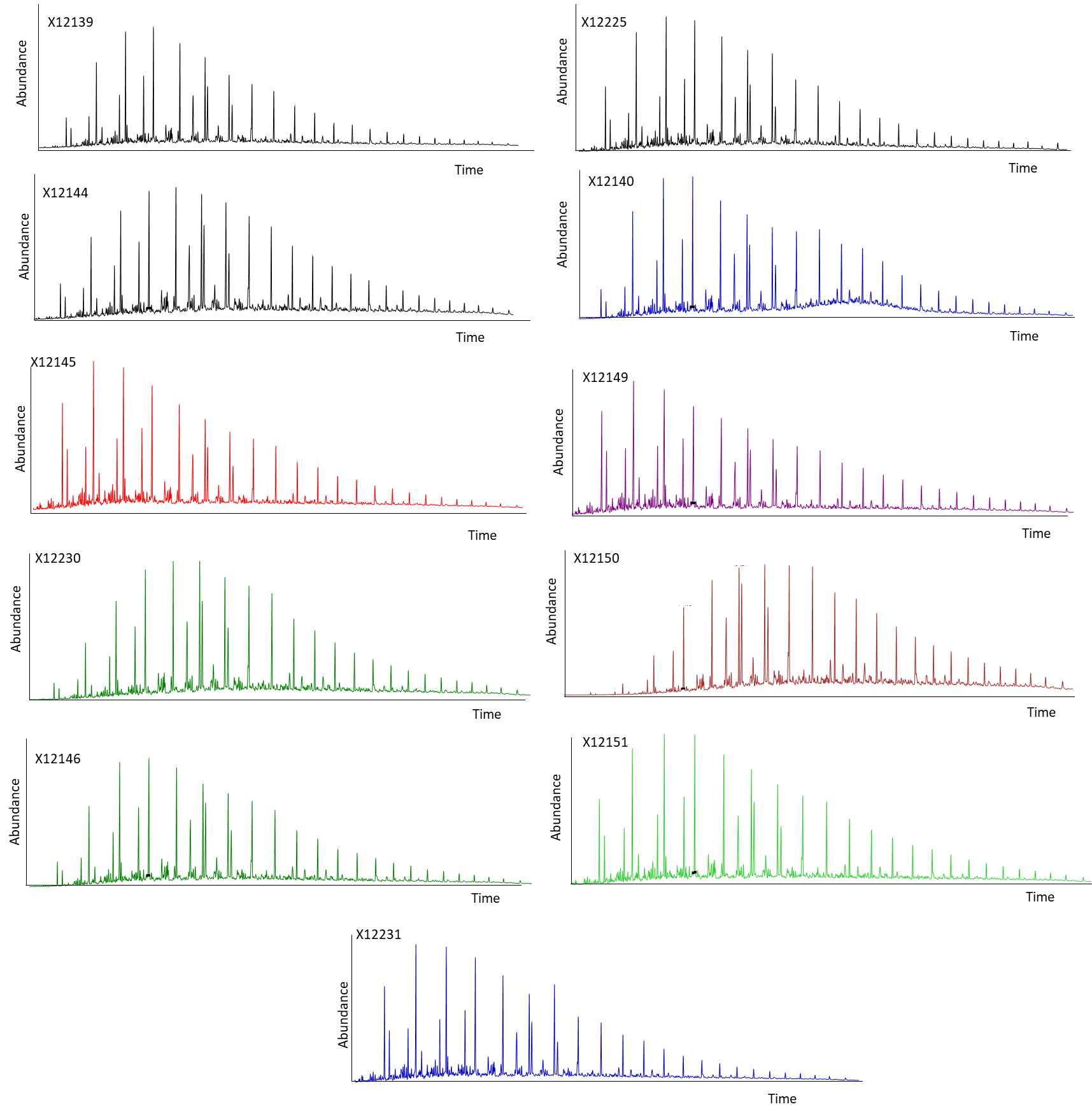


**Figure S8**. GC-FID chromatograms of the saturate fractions of the samples from this study.

**References**

Davies, G.R., 1997. The Triassic of the Western Canada Sedimentary Basin: tectonic and stratigraphic framework, paleogeography, paleoclimate and biota. Bull. Can. Petrol. Geol. 45, 434–460.

Davies, G.R., Watson, N., Moslow, T.F., Maceachern, J.A., 2018. Regional subdivisions, sequences, correlations and facies relationships of the lower Triassic Montney Formation, west-central Alberta to northeastern British Columbia, Canada — with emphasis on role of paleo structure. Bull. Can. Petrol. Geol. 66, 23–92.

Zonneveld, J.P., Moslow, T.F., 2018. Palaeogeographic setting, lithostratigraphy, and sedimentary framework of the lower Triassic Montney Formation of western Alberta and northeastern British Columbia. Bull. Can. Petrol. Geol. 66, 93–127.

McLeay Geological Consultants. Artek et al hz Inga C16-10-88-23 100/15-15-088-23W6/00. 75 p (2006).
